# Multi-echo fMRI replication sample of autobiographical memory, prospection and theory of mind reasoning tasks

**DOI:** 10.1038/sdata.2016.116

**Published:** 2016-12-20

**Authors:** Elizabeth DuPre, Wen-Ming Luh, R. Nathan Spreng

**Affiliations:** 1 Laboratory of Brain and Cognition, Human Neuroscience Institute, Department of Human Development, Cornell University, Ithaca, New York 14853, USA; 2 Cornell MRI Facility, Cornell University, Ithaca, New York 14853, USA

**Keywords:** Brain imaging, Functional magnetic resonance imaging, Cognitive neuroscience, Consciousness

## Abstract

The default network is involved in self-generated thought, a class of cognition that includes autobiographical memory, prospection, and reasoning about the mental states of others. We collected a replication sample of Spreng and Grady (*J Cogn. Neurosci*. 22, 1112–1123, 2010), confirming that the default network differentially supports each of these forms of self-generated thought. Here we describe this dataset of multi-echo fMRI data in 31 young adults during autobiographical remembering, imagining, and mentalizing; we also provide an additional resting-state scan for each subject. In this new sample, the findings from the original report are successfully replicated using the same analysis. Physiological measures were additionally collected and allow for interrogation of the impact of multi-echo independent components preprocessing both in task and rest. Future work on this dataset may provide insight into evoked brain response for cued self-generated thought, International Affective Picture System viewing, resting state dynamics, preprocessing procedures, and more. The dataset is accompanied by experimental code for independent behavioral data collection.

## Background & Summary

The default network is an active cognitive network engaged across a range of tasks related to self-generated thought^1^. This network, composed of the posterior cingulate, medial prefrontal cortex, lateral parietal cortex, and medial and lateral temporal lobes, was first delineated for its suppression during externally oriented attentional tasks^2,3^, but was also noted for its cognitive involvement in mental inference^4^. Buckner and Carroll^5^ suggested a broader role for the default network, and hypothesized that the network supported ‘self-projection’. Self-projection was described as a form of self-generated, stimulus independent thought, and included such cognitive processes as autobiographical remembering and prospection (projecting the self into the past and future, respectively), navigation (projecting the self in space) and theory of mind reasoning (projecting the self into the minds of other people).

A subsequent meta-analysis of the neuroimaging literature provided initial empirical support for the default network as the common neural substrate underlying these forms of self-projection^6^. Meta-analysis did not, however, provide the critical test of the default network’s role in self-projection seen in a within-subject design, with subjects performing multiple tasks rather than comparing results across distinct samples. To address this, Spreng and Grady^7^ developed a novel paradigm combining autobiographical memory, prospection and theory of mind conditions, and identified both common and distinct patterns of activation within the default network. Here we share data supporting a replication of this high impact neuroimaging experiment in a new subject cohort using a novel, multi-echo neuroimaging sequence. We additionally provide a multi-echo resting-state scan in the same subjects to allow for independent default network identification and comparison of network features across task and rest.

Multi-echo fMRI has been developed as a data acquisition sequence to facilitate removal of noise components from task and resting fMRI datasets^8^. This method relies on the acquisition of multiple echoes, allowing direct measurement of T2* relaxation rates. Blood-oxygen level dependent (BOLD) signal can be then distinguished from non-BOLD noise on the basis of echo time (TE) dependence. The preprocessing approach developed by Kundu and colleagues^8^ for multi-echo acquisitions is referred to as multi-echo independent components analysis (ME-ICA). ME-ICA has proven effective in denoising BOLD signal of motion and physiological artifacts both in resting state fMRI^9^ as well as in task-based^10^ fMRI experiments.

In the present dataset, the protocol developed by Spreng and Grady^7^ was replicated using multi-echo fMRI acquisition. This paradigm provides a unique opportunity to fully interrogate the denoising capabilities of ME-ICA. One relevant denoising capability of ME-ICA is its ability to account for physiological artifacts. Physiological artifacts are of particular concern in identifying dissociable correlates within the default network, where respiration and heart rate have been shown to preferentially impact connectivity patterns^11,12^. We therefore collected heart rate and respiration data for all subjects, enabling direct comparisons of ME-ICA and conventional physiological artifact correction. Additionally, ME-ICA denoising increases the signal-to-noise ratio of BOLD data^9^, allowing for the observation of subtle yet reliable patterns of functional brain response. Using multi-echo, Lombardo and colleagues^10^ recently observed cerebellar involvement in a mentalizing task that engaged the default network. The cerebellum has rarely been reported as a node of the default network, potentially due to the fact that many studies do not acquire data from the cerebellum in its entirety, although there is increasing evidence for the involvement of cerebellar regions in other large-scale brain networks^13^. It is therefore likely that the increased sensitivity of ME-ICA processed data allowed for the detection of this effect, and promises to allow for similar discovery science in the current dataset.

Here we present data collected as part of a replication study of Spreng and Grady^7^ using a novel multi-echo acquisition sequence in a new cohort of young, healthy subjects (original *n*=16; replication *n*=31). An open-access task-based multi-echo dataset could be used to investigate the effect of preprocessing and analytic strategies on results derived using multi-echo independent components analysis (ME-ICA) and multi-echo independent components regression (ME-ICR). The inclusion of a multi-echo resting state scan, in addition to being valuable in its own right, can also provide independent validation of task-driven network engagement. This dataset contributes high quality estimates of neural activation during cued self-generated thought, allowing researchers to identify activity patterns shared across tasks (c.f. ref. 14), including self-generated thought, the viewing of photographs, and resting state dynamics.

## Methods

### Participants

31 participants (16 women) were recruited from the Ithaca, New York area. All participants were right-handed young adults ranging in age from 18 to 30 years (*M*=22, *SD*=3.25) with normal or corrected-to-normal vision. Participants were screened for current psychotropic medication usage and provided written informed consent as approved by the Institutional Review Board of Cornell University.

### Task design

Task design was nearly identical to Spreng and Grady^7^ with the inclusion of an additional control condition (described below). The task consisted of three experimental conditions and two control conditions presented in four 8m40s runs. For the autobiographical memory, prospection and theory of mind conditions, the trial structure consisted of three phases: (1) a four second presentation of a stimulus, (2) a 10 s window in which a condition-specific prompt was visible, and (3) a two second fixation period. Participants were presented with photographs from the International Affective Picture System (IAPS)^15^ and asked to engage in cued autobiographical remembering, prospection, or theory-of-mind reasoning. Participants were then asked to rate the clarity of their recollection or imagined event on a scale of 1=Very Clearly to 3=Not Clearly at All. Levels of vividness were recorded as a measure of task compliance. In one control condition, participants were presented with a scrambled image for four seconds and then asked to engage in a cued button press during the subsequent ten second window to account for sensorimotor stimulation; a two second fixation period followed. In a novel second control condition, participants were presented with an IAPS image for four seconds followed immediately by a fixation cross that remained on screen for 12 s. Participants had been instructed to clear their mind when presented with a fixation cross, such that this second control condition provided an effective control for complex scene presentation in the absence of cued self-generated thought, effectively providing a null trial.

**Figure 1:**
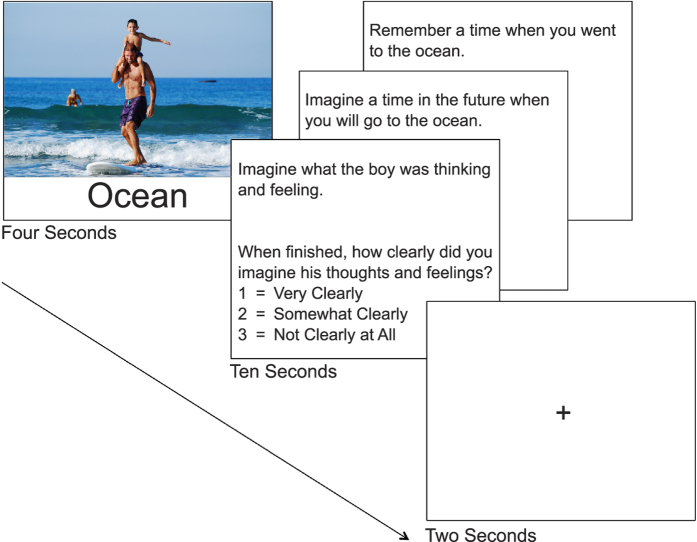
Schematic of experimental trial design. A stimulus is presented on screen for four seconds, displaying a scene and associated cue word. Here, we provide a representative, non-IAPS image (in the public domain) and cue word for illustrative purposes. Then, a condition specific prompt is presented on screen for ten seconds, during which participants are instructed to engage in the cued form of self generated thought. When finished, participants are asked to rate the clarity of their visualizations on a 1–3 scale. Two seconds of fixation separate trials without jitter.

In all conditions, stimuli presented were drawn from those in Spreng and Grady^7^. Briefly, 93 IAPS pictures without graphic depictions of nudity or mutilation were matched on valence (*M*=5.45, *SD*=1.76), arousal (*M*=4.51, *SD*=0.87), and dominance (*M*=5.42, *SD*=0.92) across experimental and relevant control conditions. Three non-IAPS images were included in the first run to increase racial diversity (available at www.github.com/lbc-spreng). All pictures were presented with an associated cue word to aid in the retrieval/constructive process. For example, in a scene showing students in a classroom, the word ‘Learning’ would be a presented. For the sensorimotor control condition, 24 scrambled images were created that matched the pictures on perceptual input and presented with the associated cue word ‘Control Stimulus’. A schematic of experimental stimuli presentation with an exemplar stimulus is presented in Fig. 1.

Within each of the four runs, six trials per condition were presented in a semi-randomized order, with run order additionally counterbalanced across participants. Prior to participation, participants were instructed on task directions, engaged in a training run, and discussed their ability to engage in the cued form self-generated thought. The instruction script read to participants appears in Supplementary File 1. Similar to Spreng and Grady^7^, participants reported no difficulties in engaging in the task after training or scanning.

### Magnetic resonance imaging

All imaging data were acquired on a 3 T GE Discovery MR750 scanner (General Electric, Milwaukee, United States) with a 32-channel receive-only phased-array head coil at the Cornell Magnetic Resonance Imaging Facility in Ithaca. All participant responses were recorded on a five-button response box held in the right hand, and responses were made using the index, middle, and ring fingers. Pulse and respiration were monitored continuously during scanning using an integrated pulse oximeter and respiratory belt. Due to a software upgrade, physiological sampling occurred at 50 Hz for 16 participants and 40 Hz for 15 participants; therefore, physiological sampling rate is provided for each participant as detailed in Data Records.

### Structural Scan

An anatomical scan was acquired during one 5m25s run using a T1-weighted volumetric MRI magnetization prepared rapid gradient echo [repetition time (TR)=2530 ms; echo time (TE)=3.4 ms; inversion time (TI)=1100 ms; flip angle (FA)=7°; bandwidth=195 Hz/pixel; 1.0 mm isotropic voxels, 176 slices]. Anatomical scans were acquired with 2x acceleration with sensitivity encoding.

### Resting fMRI

Participants completed two resting-state multi-echo BOLD functional scans. Participants were instructed to keep their eyes open, blinking and breathing normally in the dimly lit scanner bay. For 27 participants, resting-state scans were acquired prior to engaging in the task; for the remaining four participants, resting-state scans were acquired after engaging in the task. Some evidence suggests that resting-state scans acquired after task may contain neural traces of the task (e.g. ref. 16). Therefore, scan acquisition order by participant is provided as detailed in Data Records. For all participants, one 10m06s eyes-open resting-state functional scan is provided with this release, and was acquired using a multi-echo echo planar imaging (ME-EPI) sequence with online reconstruction [TR=3000 ms; echo time TE=13.7, 30, and 47 ms; flip angle FA=83°; matrix size=72×72; field of view (FOV)=210 mm; 46 axial slices; 3.0 mm isotropic voxels; slice order=inferior-superior interleaved]. Resting-state functional scans were acquired with 2.5x acceleration with sensitivity encoding.

### Task fMRI

Four 8m40s experimental runs of BOLD functional scans were acquired with a ME-EPI sequence with online reconstruction [TR=2000 ms; TE=13, 27, and 43 ms; FA=77°; matrix size=64×64; FOV=240 mm; 33 axial slices; slice thickness 3.8 mm; slice order=inferior-superior interleaved]. Functional scans were acquired with 2x acceleration with sensitivity encoding. Differences between the resting and task fMRI protocol in TR, TE and coverage may impact direct comparisons.

### Data collection quality assurance and control

A number of measures were taken to ensure reliable high quality data collection. All participants were informed about the importance of staying still during the MR scan. Task requirements were explained thoroughly prior to scanning by a trained research assistant working from a script (see Supplementary File 1) to ensure understanding of the task demands and procedure. This included pre-scan practice trials. The research assistant oversaw the implementation of the experimental procedure, including confirmation that the participant could see the display, button box training and testing on placement in the scanner, and button press confirmation just prior to the first experimental run. All scans at the Cornell Magnetic Resonance Imaging Facility were performed by a trained MR technician working with a standardized protocol. This ensured consistent data acquisition procedures, including visual checks for coverage, ongoing quality assessment, and confirmation of participant wakefulness between runs. Post-scan quality assurance is discussed below.

### Code Availability

Experimental code was created using EPrime software version 2.0.10.242 (www.pstnet.com) and is freely available at www.github.com/lbc-spreng. We do not have permission to distribute the IAPS provided stimuli; therefore, researchers must obtain the IAPS dataset to run the posted experimental code. This can be done via a confirmed IAPS User Agreement with the Center for the Study of Emotion and Attention at the University of Florida (http://csea.phhp.ufl.edu/). Non-IAPS images with associated cue words are available at the listed repository.

## Data Records

All data listed in this section are available on OpenfMRI (Data Citation 1). A README file with a detailed description of the data is also available at this URL.

### Demographics

Location: participants.tsv

File format: plain text, tab-separated values

Basic demographic information including gender, age, race, and ethnicity is available. Due to a software upgrade during scanning, physiological recordings occurred at either 40 or 50 Hz. Physiological sampling rate is therefore included for each participant. Four participants had resting-state functional scans collected following task participation. Scan acquisition order is therefore detailed for each participant, with 1 indicating resting-state functional scans preceding task participation. All demographic information is available as a tab-separated value (TSV) file.

### Anatomical scans

Location: sub-<ID>/anat/sub-<ID>_T1w_defaced.nii.gz

File format: NIfTI, gzip-compressed

MRI data are available in NIfTI file format. All structural scans have been defaced as part of the de-identifying process. The defacing procedure was performed following DICOM to NIfTI conversion using AFNI’s to3d command; we therefore do not provide DICOM files.

### Resting-state functional scans

Location: sub-<ID>/func/sub-<ID>_task-rest_run-01_bold.e0[1-3].nii.gz

File format: NIfTI, gzip-compressed

### Functional scans

Location: sub-<ID>/func/sub-<ID>_task-cuedSGT_run0[1-4]_bold.e0[1-3].nii.gz

File format: NIfTI, gzip-compressed

### Physiological recordings

Location: sub-<ID>/func/sub-<ID>_task-[cuedSGT|rest]_run0[1-4]_physio.tsv.gz

File format: plain text, tab-separated values

Physiological recordings for respiration and heart rate are provided as a tab-separated value (TSV) file.

### Behavioral responses

Location: sub-<ID>/func/sub-<ID>_task-cuedSGT_run0[1-4]_events.tsv

File format: plain text, tab-separated values

For each trial of the cued self-generated thought task, participant responses representing clarity ratings and sensorimotor control button presses are provided with corresponding reaction times. Additional trial wise information including IAPS identifier of the presented stimulus, associated cue word, trial condition, and timing (i.e., onset and duration) is also available. All behavioral information is available as a tab-separated value (TSV) file.

## Technical Validation

### Replication of previous results

**Figure 2:**
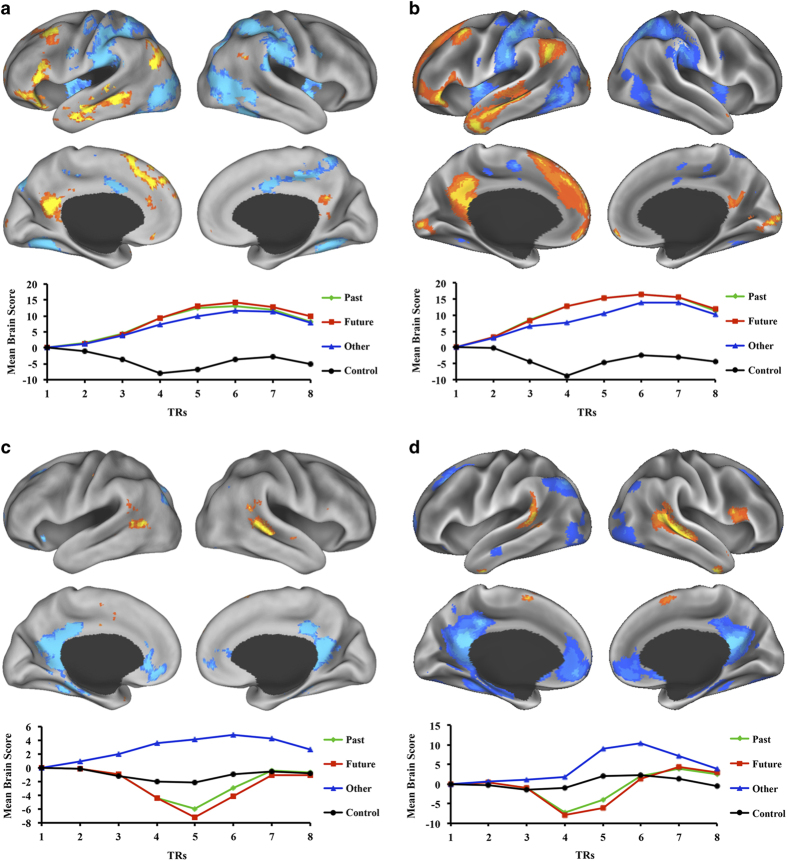
Original and replication sample results. The first latent variable for the (**a**) original (*P*=0.002)^7^ and (**b**) replication sample (*P*=0.001). For (**a**,**b**), warm colors show patterns of covarying activity for autobiographical memory (Past), prospection (Future) and theory of mind (Other). Cool colors depict the control condition. The second latent variable for the (**c**) original (*P*=0.056) and (**d**) replication sample (*P*=0.009). For (**c**,**d**), autobiographical memory and prospection (represented as cool colors) are dissociated from theory-of-mind (warm colors), where control is roughly at zero. For each latent variable, temporal brainscores (summed scores of activity across the entire brain of each participants and averaged across participants) show the divergence between conditions over time (eight 2-sec TRs). The results derived from the current replication, shown in **b**,**d**, closely resemble the original results presented in **a**,**c**. The spatial extent of the replicated results is more extensive than those seen in the original publication and includes some regions outside of the default network.

To confirm that the current dataset replicated previous results reported by Spreng and Grady^7^, data were preprocessed with ME-ICA^8^ version 3.0 (https://bitbucket.org/prantikk/me-ica). Anatomical images were first skull stripped using the default parameters in FSL BET. ME-ICA processing was then run with the following options: -e 12.8, 27.5, 43.0; -b 4v; --fres=2.0; --no_skullstrip; --qwarp; –space=MNI_caez_N27+tlrc. Finally, ME-ICA denoised time series were smoothed with 8mm FWHM using AFNI *3dmerge*, and submitted to Partial Least Squares (PLS)^17,18^. Resulting statistical maps and temporal brainscores for the two significant latent variables are presented in Fig. 2 alongside original results from the Spreng and Grady^7^ publication. The unthresholded statistical maps are available through NeuroVault: http://neurovault.org/collections/1866/. These results confirm the replicability of the original effect.

### Clarity ratings

**Figure 3:**
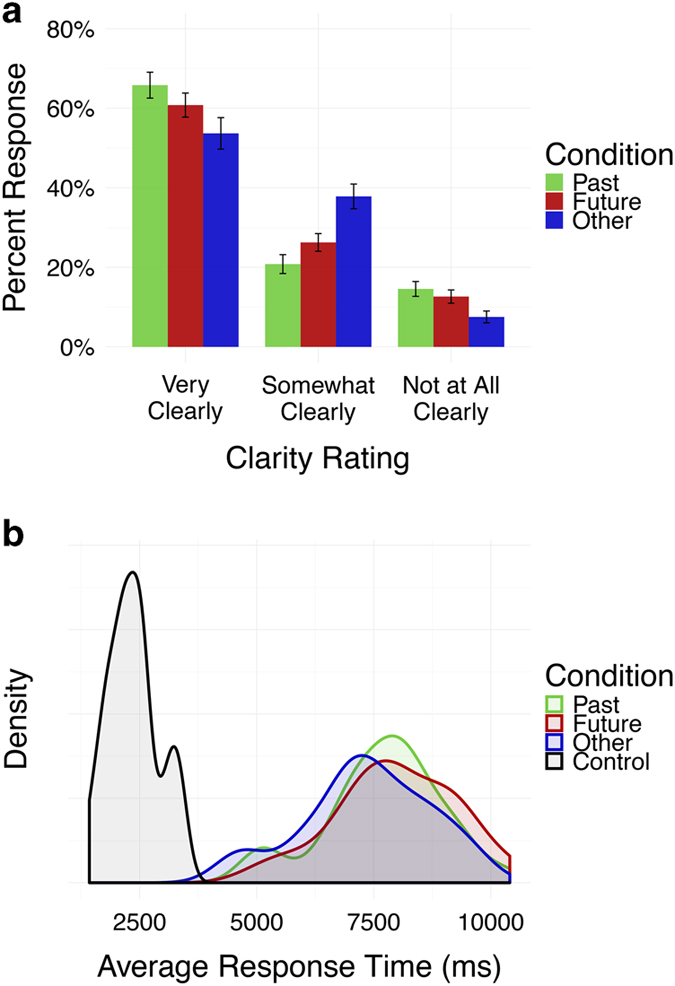
Behavioral results. (**a**) Percentage of responses for each clarity rating in each cued self-generated condition. On average, participants self-reported high levels of clarity for all three conditions. Statistical testing showed no significant differences in clarity ratings between the three conditions. (**b**) The distribution of average responses times for each experimental condition. The Control condition elicited significantly faster reaction times than the experimental conditions, which did not differ from each other.

To assess self-reported vividness for each form of cued self-generated thought, clarity ratings were examined by self-generated thought condition (i.e., autobiographical remembering, prospection, and theory of mind reasoning; See Fig. 3a). There was no significant difference in the means of self-reported clarity across conditions, F(2,60)=0.4, *P*=n.s. Mean clarity ratings were high for each condition, suggesting that participants were able to reliably and vividly engage in each form of self-generated thought.

### Reaction times

To describe the distribution of response times for the sensorimotor control and cued self-generated thought conditions, reaction times were plotted by experimental condition (See Fig. 3b). Reaction times for the sensorimotor control condition were concentrated at 2.5 s, while reaction times for cued forms of self-generated thought occurred later, with a majority between 7–8 s. Subsequent statistical testing confirmed a significant difference in reaction time by condition, F(3,90)=326.4, *P*<0.001. A post-hoc Tukey test (α=0.05) showed that reaction times for the control condition were significantly faster than the cued self-generated thought conditions, which did not differ from one another. These results suggest that participants were engaged in the task and that participants were able to hold in mind each form of cued self-generated thought.

### Magnetic resonance imaging

To assess fMRI scan quality, quality metrics were calculated for each scan.

- **Framewise Displacement (FD).** A measure of the frame-to-frame movement, assessed in millimetres. FD was calculated with the six-parameter motion file output by meica.py separately for task and resting-state scans. For task scans, the average FD was 0.11 mm (*SD*=0.06); for resting-state scans, the average FD was 0.08 mm (*SD*=0.04 mm). FD values greater than 0.20 mm are conventionally considered high motion^19^; we therefore also calculated the percentage of frames for each subject where FD exceeded 0.20 mm. For task scans, the average percentage of frames where FD was greater than 0.20 mm was 10% (*SD*=14%); for resting-state scans the average percentage was 6% (*SD*=8%).
- **Temporal Signal to Noise Ratio (tSNR).** A measure of signal strength at the voxel level, calculated as the mean signal intensity of a voxel across the timeseries divided by its standard deviation. tSNR was calculated with three preprocessing approaches: 1) the middle echo image, as this most closely approximates standard single echo collection, 2) an optimal combination, and 3) an optimal combination with the ME-ICA denoising. Image files were first high-pass filtered with *f*>0.02 to remove low frequency drift using AFNI *3dFourier*. Following Kundu *et al.*^9^, tSNR was then calculated only within the overlap of a grey matter and functional mask. The grey matter mask was calculated by resampling a skullstripped image to functional resolution and segmenting using the FAST segmentation algorithm implemented in FSL. The functional mask was calculated from the functional data using the 3dAutomask function implemented in AFNI. The median of all voxels within this mask is used to characterize each participant’s scan, where higher tSNR values reflect clearer signal.

**Figure 4:**
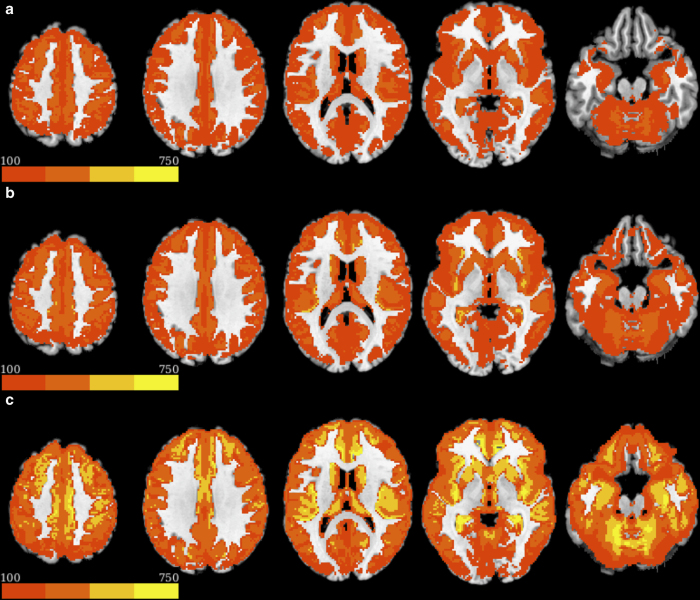
Impact of single versus multi-echo and preprocessing on temporal SNR. tSNR was calculated for the (**a**) minimally preprocessed second echo, (**b**) optimally combined timeseries, and (**c**) the ME-ICA denoised optimally combined timeseries. Group-average tSNR maps were thresholded at 100. There is a clear increase in tSNR from the minimally processed single-echo data (which is analogous to standard single echo acquisition), to the optimal combination of echoes and is best with the optimal combination of echoes and ME-ICA denoising. Improvement in spatial coverage can also be observed, with regions such as orbital frontal cortex showing acceptable levels of tSNR.

In order to investigate the effects of ME-ICA denoising on signal quality in the present dataset, tSNR was calculated on minimally preprocessed and ME-ICA denoised data. tSNR for the motion-corrected and spatially-aligned second echo, the optimal combination of minimally preprocessed echoes, and the ME-ICA denoised timeseries was derived as described above. Derived tSNR spatial maps were averaged across all subjects, thresholded at 100, and plotted in Fig. 4. The results show a clear increase in tSNR both with the optimal combination of echoes and ME-ICA denoising compared to the standard single-echo acquisition.

## Additional Information

**How to cite this article**: DuPre, E. *et al.* Multi-echo fMRI replication sample of autobiographical memory, prospection and theory of mind reasoning tasks. *Sci. Data* 3:160116 doi: 10.1038/sdata.2016.116 (2016).

**Publisher’s note**: Springer Nature remains neutral with regard to jurisdictional claims in published maps and institutional affiliations.

## Supplementary Material



Supplementary File 1
