# Supplementary material for: Multi-echo fMRI replication sample of autobiographical memory, prospection and theory of mind reasoning tasks
Source: Sci Data. 2016 Dec 20;3:160116. doi: 10.1038/sdata.2016.116 (PMC5170594; doi:10.1038/sdata.2016.116)
Supplement: Supplementary File 1 [file sdata2016116-s2.doc]

***Supplementary File 1***

**Instruction Script**

You will be seeing a number of photographs with a related word underneath it. Please read each word and examine each photograph. It will be presented for 4 seconds.

Following the photo, you will be presented with one of three questions.

In some, you will be asked to remember a single and specific event from your past. In others, you will be asked to image a highly probable event in your own personal future. Finally, some questions will ask you to imagine the thoughts and feelings of the people in the photograph you just saw. Concentrate on imagining the scene, including what it looks and feels like.

If a memory, try to remember an event that is specific in place and time. The event can span a few minutes or maybe even a few hours, but not many hours or days. Try to be very specific and recall what the environment looked like, what you thought and felt at the time you experienced the event.

If a future event, imagine the context you will be in and how it makes you feel, what will be happening, how you are there. Try to imagine a complete and coherent scene.

When thinking about others, try to vividly imagine the context, thoughts, and feelings of the people in the photograph. Try to put yourself in their shoes.

You will be asked to rate the clarity of this experience on a scale of one to three. Clarity can relate to many things, such as how vividly you “see” the event. Additionally, clarity also includes how coherently you experience it, how it feels and how clear your thinking is. A rating of “three” would indicate that you could not remember anything at all, while a “one” would be a very vivid re-creation in your memory. For future events, a rating of “three” would be no event and a “one” would be the envisioning of a clear event in the future that you are involved in. When imagining the thoughts and feelings of others, a rating of “three” would indicate you could not imagine the person’s thoughts or feelings at all; a “one” would suggest a good deal of experiencing their emotion, imagining their thoughts, or placing yourself in their environment. A rating of “two” is in the middle.

In general, if you cannot think of anything, give it a 3. If it is moderate, a 2. If you really have a good sense of it, give it a 1.

You will have 10 seconds for this phase. Please respond as the 10 seconds nears the end. Don’t worry too much about timing. You will get the hang of when to respond.

Sometimes a photo will be shown, followed by a “+”. For this, just clear your mind and relax.

In addition to these photos, you will also see some scrambled images. For these, simply press the button you are instructed to.

In all cases, please take your time to respond. You are not judged for speed. We are more interested in how much you engage in the task.

You will be asked to think about a number of things while you are scanned. It is important that you try to clear your mind and relax when you see the “+” and proceed to the next image. You will get a brief break in between sessions.
